# High *TXLNA* Expression Predicts Favourable Outcome for Pancreatic Adenocarcinoma Patients

**DOI:** 10.1155/2020/2585862

**Published:** 2020-02-25

**Authors:** Shuangyu Lv, Guosen Zhang, Longxiang Xie, Zhongyi Yan, Qiang Wang, Yongqiang Li, Lu Zhang, Yali Han, Huimin Li, Yaowu Du, Yanjie Yang, Xiangqian Guo

**Affiliations:** Department of Preventive Medicine, Institute of Biomedical Informatics, Cell Signal Transduction Laboratory, Bioinformatics Center, Henan Provincial Engineering Center for Tumor Molecular Medicine, School of Basic Medical Sciences, Henan University, Kaifeng 475004, China

## Abstract

TXLNA (taxilin alpha), a binding partner of the syntaxin family, was identified as a key factor in the coordination of intracellular vesicle trafficking and highly expressed in various tumor cells. However, the accurate relation between TXLNA and tumorigenesis and progression of pancreatic adenocarcinoma (PAAD) is still unclear. The present study was designed to examine the expression profile of TXLNA and explore its prognostic significance in PAAD patients and the possible molecular regulatory mechanism by analyzing a series of data from databases, including GEPIA, LOGpc, STRING, and GeneMANIA. The results indicate that TXLNA mRNA and protein were remarkably increased in PAAD tissues compared with normal pancreatic tissues. The high TXLNA expression was significantly correlated with superior overall survival (OS), disease-free interval (DFI), disease specific survival (DSS), and progression-free interval (PFI) for PAAD patients. In summary, high TXLNA expression could predict favourable OS, DFI, DSS, and PFI for PAAD patients, and it might be as potential prognostic biomarkers and targets for PAAD.

## 1. Introduction

Pancreatic cancer (PC) is one of the leading causes of cancer mortality, resulting in a substantial global burden [1]. It has become the third leading cause of cancer death in the USA, with a 5-year survival rate of 8% [2]. In recent decades, the treatment of pancreatic cancer has been improved; however, the diagnostic and prognostic biomarkers for pancreatic cancer are still deficient due to its heterogeneous characteristics [3]. Up to now, carbohydrate antigen 19-9 (CA19-9) is the only available diagnostic biomarker for pancreatic adenocarcinoma (PAAD) approved by the Food and Drug Administration (FDA) in the U.S., but it had a limitation of poor sensitivity and specificity [4, 5]. Therefore, it is urgent to search for novel biomarkers.

Taxilin alpha (TXLNA) was identified as a binding partner of the syntaxin family [6]. The TXLNA was also named interleukin 14 (IL-14), and its alternative gene name was designated as *TXLNA* or *IL14* in the NCBI. The function of TXLNA was identified as a key factor in the coordination of intracellular vesicle trafficking [6]. The syntaxin family was composed of three members, including alpha (*α*)-, beta (*β*)-, and gamma (*γ*)-taxilin. In addition, they share an extraordinarily long coiled-coil region homologous to that of Uso1, a yeast tethering factor homolog of p115 [7]. It was reported that the syntaxin family involved in the transport of the vesicles delivered to the plasma membrane [6, 7]. As we know, the intracellular vesicle tranfficking is a primary transportation system in eukaryotic cells, and vesicle tranfficking participates in the multiple processes of cell proliferation. The abnormal proliferation indicates a high probability of tumor or cancer [8].

Recent studies demonstrate that the TXLNA protein had a high expression in various tumor cells and its expression indicates a close relationship with histological grade and proliferative activity in hepatocellular carcinoma, as well as the metastatic and invasive potential of renal cell carcinoma [8–11]. Sakakibara et al. showed that a strong expression of TXLNA was detected in proliferating neuroepithelial cells during embryonic development of the rat brain [12]. Horii et al. have found that the expression of TXLNA was correlated with cell proliferation in the adult murine gastrointestinal tract and during postnatal development [9].

The relationship between TXLNA and pancreatic cancer prognosis remains to be determined. The present study was designed to evaluate the expression profile of TXLNA and to investigate its prognostic significance for PAAD patients.

## 2. Materials and Methods

### 2.1. TXLNA Gene Expression Analysis

The TXLNA mRNA level in PAAD tissue and normal pancreatic tissue was examined by on-line analysis GEPIA (Gene Expression Profiling Interactive Analysis) (http://gepia.cancer-pku.cn). GEPIA is a web server which analyzes the RNA expression data of 9,736 tumors and 8,587 normal samples from TCGA and GTEx [13].

### 2.2. Prognosis Analysis

The relationship between the expression of TXLNA and survival terms, including overall survival (OS), disease-free interval (DFI), disease specific survival (DSS), and progression-free interval (PFI), in 542 PAAD patients was determined by the prognosis analysis web server LOGpc [14] (Long-term Outcome and Gene Expression Profiling Database of pan-cancers, http://bioinfo.henu.edu.cn/DatabaseList.jsp) using TCGA and ICGA_Seq as we previously reported [15]. This LOGpc tool encompasses 171 expression datasets and provides 13 types of survival terms for 27929 patients of 26 distinct malignancies.

### 2.3. Gene Interaction Network Construction

Search Tool for the Retrieval of Interacting Genes (STRING, version 11.0, available online: http://www.string-db.org/) is an open-source database, which was applied to predict protein-protein interaction (PPI) networks of differentially expressed genes (DEGs) [16]. In addition, only the interactions with a combined score >0.4 were considered to have a statistically significant difference. The present new version contains 5090 organisms, 24584628 proteins, and 3123056667 total interactions. GeneMANIA (http://genemania.org/) was utilized to conduct analysis of TXLNA correlated genes. GeneMANIA is a prediction server to search for other genes related to the targeted genes using analysis of functional association, including protein and genetic interactions, pathways, coexpression, colocalization, and protein domain similarity [17].

### 2.4. Statistical Analysis

Univariate and multivariate Cox regression models were utilized to assess the prognostic role of TXLNA in terms of OS, DFI, PFI, and DSS for PAAD patients using SPSS. Risk factors (*p* < 0.05) analyzed by univariate analysis were selected for multivariate Cox regression analysis. A value of *p* < 0.05 was considered statistically significant.

## 3. Results

### 3.1. TXLNA Expression in PAAD Tissue Was Increased Compared with Normal Tissue

**Figure 1:**
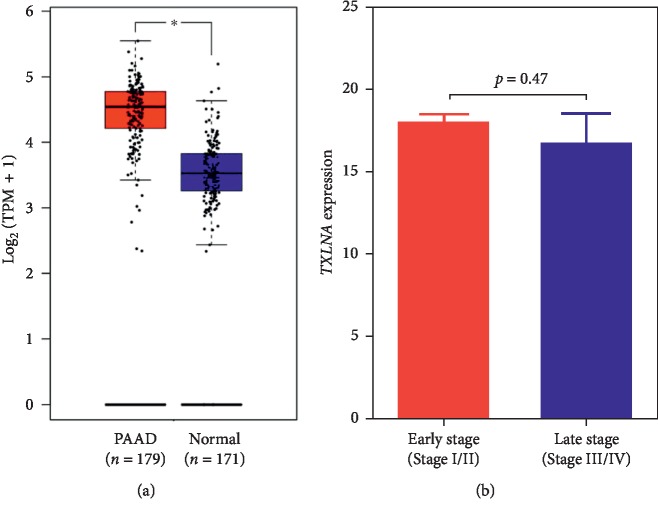
The *TXLNA* gene expression in pancreatic adenocarcinoma (PAAD) tissue and normal pancreas tissue. (a) The *TXLNA* expression in tumor tissue and normal tissue was analyzed using Gene Expression Profiling Interactive Analysis (GEPIA). (b) The *TXLNA* expression at early and late clinical stages of PAAD cases was analyzed based on the TCGA dataset. TPM, transcripts per million reads.

As shown in Figure 1(a), the GEPIA analysis based on TCGA and the GTEx data indicates that PAAD tissues exhibited a significantly increased TXLNA expression, compared with the normal tissue (*p* < 0.05). There was no significant difference of TXLNA expression between the TNM stages (*p*=0.47, Figure 1(b)) and different neoplasm histologic grades (*p*=0.267, [Supplementary-material supplementary-material-1]).

### 3.2. High TXLNA Expression Was Correlated with Favourable OS, DFI, DSS, and PFI in PAAD Patients

**Figure 2:**
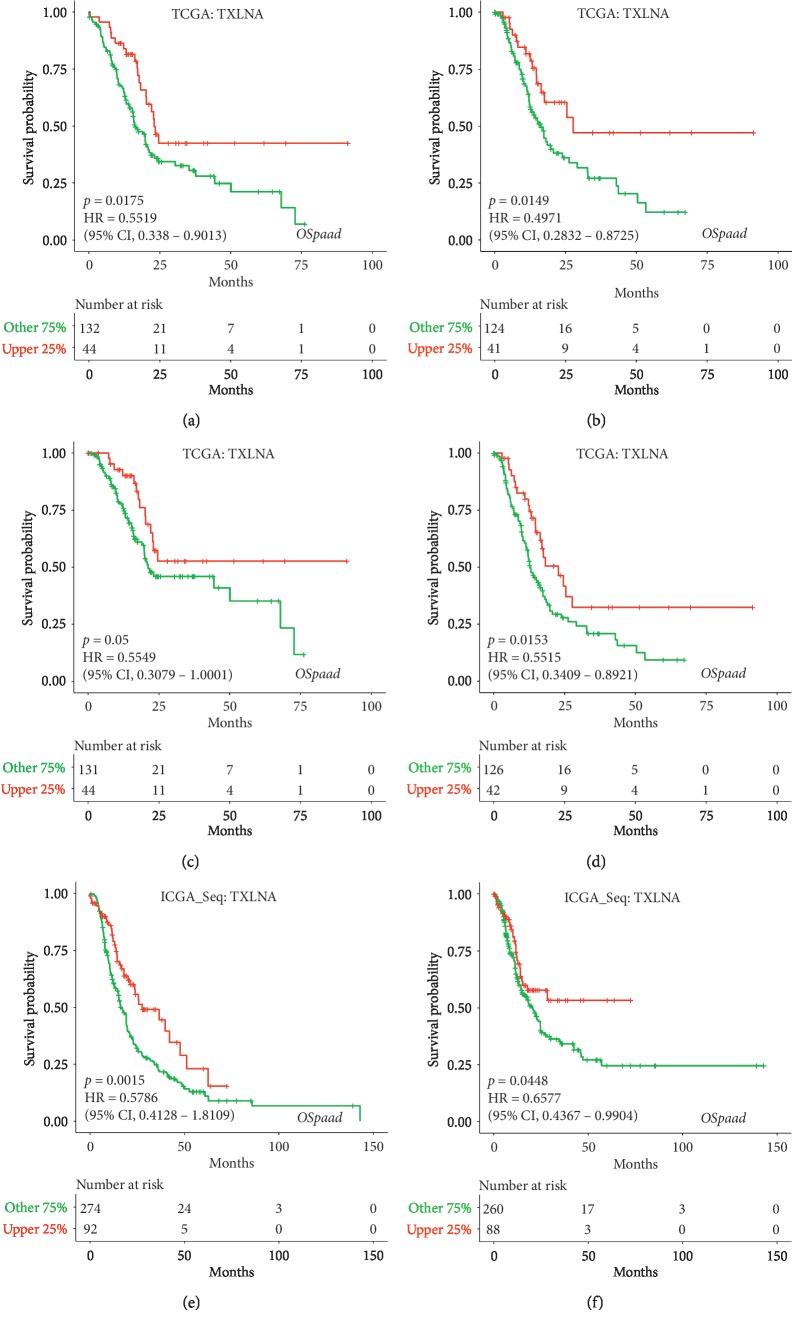
Kaplan-Meier curves of overall survival (OS) (a), disease-free interval (DFI) (b), disease specific survival (DSS) (c), and progression-free interval (PFI) (d) from TCGA data in PAAD. Kaplan-Meier curves of overall survival (OS) (e) and disease-free interval (DFI) (f) from ICGA data in PAAD.

The prognostic value of TXLNA was tested by KaplanMeier Plotter Analysis of LOGpc [18, 19]. Analysis based on TCGA data in LOGpc showed that the high *TXLNA* expression group had favourable OS (*p*=0.0175; HR: 0.5519; 95% CI: 0.338–0.9013; Figure 2(a)), DFI (*p*=0.0149; HR: 0.4971; 95% CI: 0.2832–0.8725; Figure 2(b)), DSS (*p*=0.05; HR: 0.5549; 95% CI: 0.3079–1.0001; Figure 2(c)), and PFI (*p*=0.0153; HR: 0.5515; 95% CI: 0.3409–0.8921; Figure 2(d)) for PAAD patients. Meanwhile, analysis results from ICGA_Seq data demonstrated that the increased *TXLNA* expression group had favourable OS (*p*=0.0015; HR: 0.5786; 95% CI: 0.4128–0.8109; Figure 2(e)) and favourable DFI (*p*=0.0448; HR: 0.6577; 95% CI: 0.4367–0.9904; Figure 2(f)) for PAAD patients. These results indicate that high TXLNA expression was significantly correlated with favourable OS, DFI, DSS, and PFI in PAAD patients.

### 3.3. Significant Correlation between the Increased TXLNA Expression and Favourable OS in PAAD Patients Was Restricted to Female Patients or Patients with Lymph Nodes, No-Smoking History, or Alcohol History

**Figure 3:**
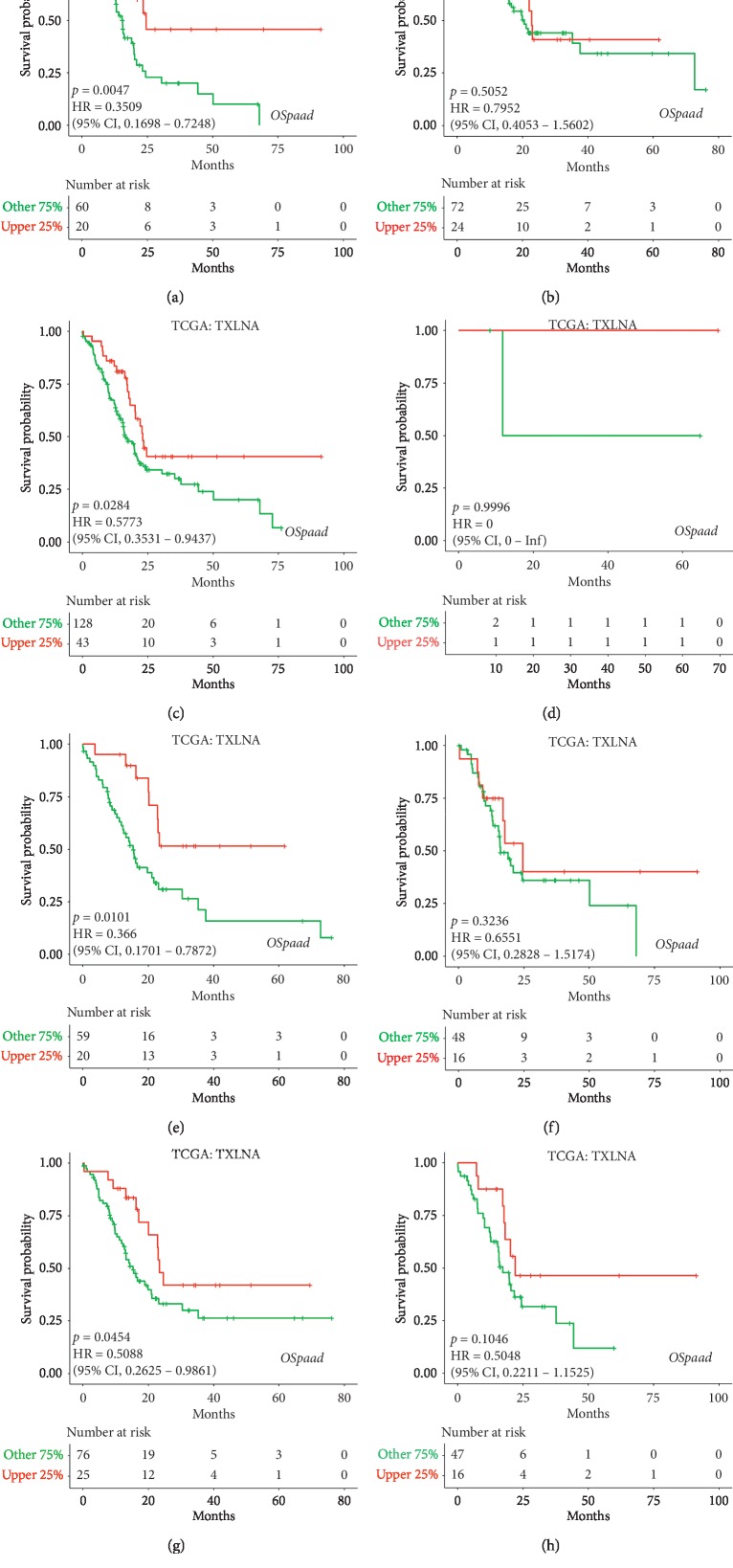
Kaplan-Meier curves of overall survival (OS) in female (a), male (b), lymph nodes (c), no-lymph nodes (d), no-smoking (e), smoking (f), alcohol (g), and no-alcohol (h) from TCGA data in PAAD.

To examine whether the correlation was influenced by the different clinical characteristics of PAAD patients, such as gender, lymph nodes, and smoking or alcohol, each character was used to divide PAAD patients into two subgroups (male and female, lymph nodes and no-lymph nodes, smoking and no-smoking, and alcohol and no-alcohol) for analysis. The high *TXLNA* expression group had favourable OS in female PAAD patients (*p*=0.0047; HR: 0.3509; 95% CI: 0.1698–0.7248; Figure 3(a)), but not in male PAAD patients (*p*=0.5052; HR: 0.7952; 95% CI: 0.4053–1.5602; Figure 3(b)). The increased *TXLNA* expression group had favourable OS in PAAD patients with lymph nodes (*p*=0.0284; HR: 0.5773; 95% CI: 0.3531–0.9437; Figure 3(c)), but not in PAAD patients with no-lymph nodes (*p*=0.9996; Figure 3(d)). The high *TXLNA* expression group had favourable OS in PAAD patients with no-smoking history (*p*=0.0101; HR: 0.366; 95% CI: 0.1701–0.7872; Figure 3(e)), but not in PAAD patients with smoking history (*p*=0.3236; HR: 0.6551; 95% CI: 0.2828–1.5174; Figure 3(f)). The increased *TXLNA* expression group had favourable OS in PAAD patients with alcohol history (*p*=0.0454; HR: 0.5088; 95% CI: 0.2625–0.9861; Figure 3(g)), but not in PAAD patients with no-alcohol history (*p*=0.1046; HR: 0.5048; 95% CI: 0.2211–1.1525; Figure 3(h)). These results demonstrated that high *TXLNA* expression indicated a favourable OS in female patients or patients with lymph nodes, no-smoking history, or alcohol history.

### 3.4. TXLNA Expression Was an Independent Prognostic Biomarker for Favourable OS, DFI, and PFI in PAAD

Univariate analysis demonstrated that the lymph nodes and increased TXLNA expression correlated with favourable OS, DFI, PFI, and DSS for PAAD (Table 1). Multivariate analysis indicated that high TXLNA expression was an independent prognostic indicator in terms of OS (HR: 1.641; 95% CI: 1.003–2.685; *p*=0.048), DFI (HR: 1.848; 95% CI: 1.051–3.250; *p*=0.033), and PFI (HR: 1.668; 95% CI: 1.029–2.702; *p*=0.038; Table 1).

### 3.5. Construction of Protein-Protein and Gene-Gene Interaction Network

**Figure 4:**
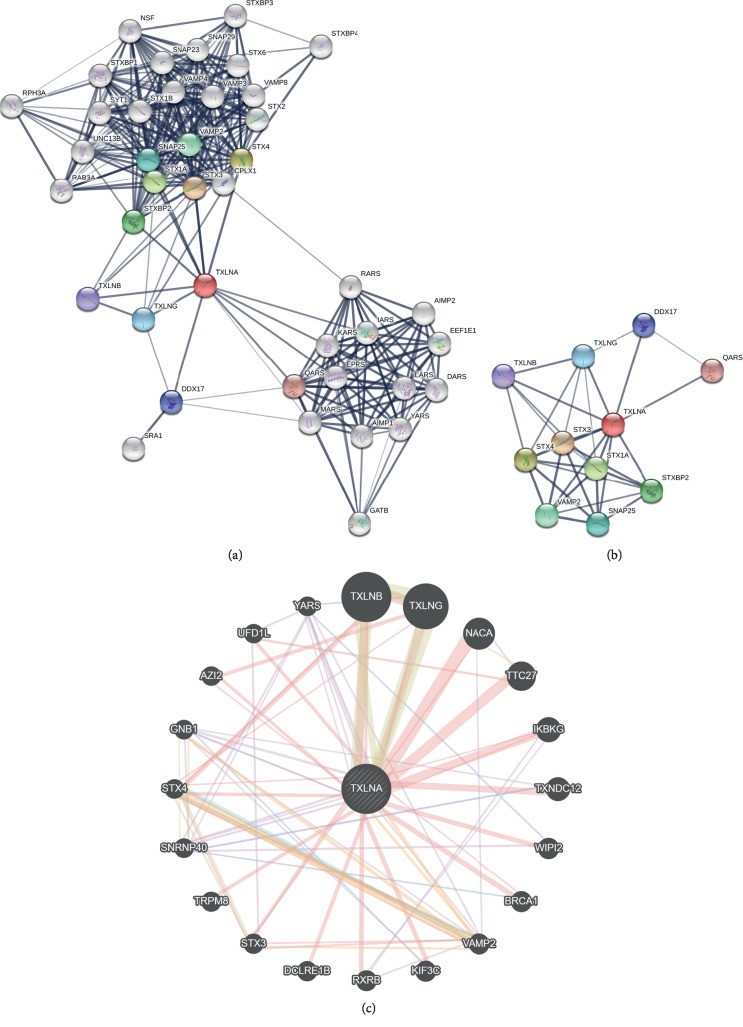
Interaction analysis of TXLNA at the gene and protein levels. (a) The PPI network constructed with STRING contained 41 nodes and 310 edges. (b) The PPI network of 10 hub proteins (34 edges) was extracted from A. The line thickness indicates the strength of data support. (c) Gene-gene interaction network for *TXLNA* was analyzed using GeneMANIA prediction server (http://genemania.org/).

To explore the potentially interacted proteins with TXLNA, the PPI network was constructed by STRING. After removing unconnected nodes, the PPI network containing 41 nodes and 310 edges was established (Figure 4(a)). To further investigate the hub proteins in the network, the top 10 hub proteins were retrieved: STX3, STX4, STX1A, STXBP2, VAMP2, SNAP25, TXLNG, DDX17, TXLNB, and QARS (Figure 4(b)). Gene-gene interaction analysis using GeneMANIA showed 20 correlated genes with *TXLNA*, such as *TXLNB*, *TXLNG*, *NACA*, *TTC27*, *IKBKG*, *TXNDC12*, *WIPI2*, *BRCA1*, *VAMP2*, and *KIF3C* (Figure 4(c)).

## 4. Discussion

Pancreatic cancer is a highly heterogeneous disease with a low overall survival rate [2, 3]. Therefore, it is essential to discover effective tumor biomarkers to improve the clinical diagnosis, prognosis, and treatment. Recently, a variety of prognostic markers for pancreatic cancer are reported. Li et al. had found that the combination of SRPX2 and RAB31 were independent prognostic factors that associated with OS and DFS of pancreatic cancer [20]. Chen et al. reported that three hypomethylated genes (*SULT1E1*, *IGF2BP3*, and *MAP4K4*) were associated with poor overall survival of pancreatic cancer patients [21]. Pang et al. have indicated that *IL22RA1*, *BCL2L1*, *STAT1*, *MYC*, and *STAT2* involved in the Jak-STAT signaling pathway may be significantly associated with prognosis of pancreatic cancer [22].

Recent studies indicate that TXLNA was involved in inflammation, endocrine, and respiratory diseases. IL-14, a cytokine, was identified as high molecular weight B-cell growth factor [23]. Its mRNA transcript level was increased in vitro in human T cells responding to alloantigen stimulation [24]. The IL-14, IL-17, and TNF-*α* level in whole lung homogenates were upregulated in RAGE transgenic mice [25]. Helen Kemp et al. had demonstrated that the thyroid follicular cells in the thyroid had the ability to express IL-14 mRNA [26]. Nevertheless, the role of TXLNA on pancreatic cancer remains unknown.

For PAAD patients, prognostic factors could help to guide the personalized treatments. The correlation of TXLNA expression and prognostic value was analyzed using LOGpc. We discovered that increased *TXLNA* expression was significantly correlated with favourable OS, DFI, PFI, and DSS in PAAD patients in TCGA data. Analysis from ICGA_Seq data also indicated that the upregulated *TXLNA* expression was significantly correlated with favourable OS and DFI. In addition, further analysis suggested that the correlation of high *TXLNA* expression and favourable OS was influenced by gender, smoking, alcohol, and lymph history in patients with PAAD. Gender was a key factor for prognostic analysis. Previous studies indicated that gender had a significant influence on the outcome of prognostic analysis, such as disease-free survival, and distant metastasis [27–29]. Our study showed that the high TXLNA expression group exhibited favourable OS in female PAAD patients, but not in male. The gender differences in the prognosis of PAAD could be explained by genetic variants affected by the hormonal environment. It suggests that high TXLNA expression was suitable for prognosis of female (not male) PAAD patients. Multivariate analysis showed that high *TXLNA* expression was an independent prognostic indicator in terms of OS, DFI, and PFI in PAAD patient.

In conclusion, TXLNA has a high expression in PAAD tissue compared with normal tissue, and its upregulated expression might serve as an independent prognostic indicator of OS, DFI, DSS, and PFI in PAAD patients. Further experimental studies are needed to fully understand the association between TXLNA expression and prognosis of PAAD patients.

## Figures and Tables

**Table 1:** Univariate and multivariate analysis of OS, DFI, PFI, and DSS in patients with PAAD in the TCGA.

Parameters	Univariate analysis				Multivariate analysis			
	*p*	HR	95% (lower/upper)		*p*	HR	95% (lower/upper)	
OS								
Age >65 vs. ≤65	0.141	0.752	0.515	1.099				
Female vs. male	0.228	0.793	0.545	1.156				
Smoking history 2/3/4/5 vs. 1	0.732	1.076	0.706	1.641				
Alcohol history Yes vs. no	0.878	1.033	0.685	1.558				
Lymph node yes vs. no	0.001	2.236	1.373	3.641	0.004	0.477	0.288	0.788
Clinical stage III/IV vs. II/I	0.807	0.894	0.363	2.202				
TXLNA expression High vs. low	0.018	1.811	1.109	2.958	0.048	1.641	1.003	2.685
DFI								
Age >65 vs. ≤65	0.287	0.787	0.507	1.222				
Female vs. male	0.661	0.908	0.591	1.396				
Smoking history 2/3/4/5 vs. 1	0.502	1.173	0.736	1.868				
Alcohol history Yes vs. no	0.382	0.810	0.504	1.300				
Lymph node Yes vs. no	0.033	0.585	0.358	0.957	0.048	0.601	0.363	0.996
Clinical stage III/IV vs. II/I	0.328	0.561	0.176	1.786				
TXLNA expression High vs. low	0.016	1.999	1.139	3.509	0.033	1.848	1.051	3.250
PFI								
Age >65 vs. ≤65	0.159	1.318	0.897	1.937				
Female vs. male	0.815	0.956	0.656	1.394				
Smoking history 2/3/4/5 vs. 1	0.786	1.059	0.7	1.602				
Alcohol history Yes vs. no	0.63	0.902	0.593	1.372				
Lymph node Yes vs. no	0.004	0.524	0.336	0.819	0.012	0.558	0.354	0.881
Clinical stage III/IV vs. II/I	0.922	1.039	0.481	2.247				
TXLNA expression High vs. Low	0.017	1.8	1.113	2.912	0.038	1.668	1.029	2.702
DSS								
Age >65 vs. ≤65	0.640	1.115	0.706	1.762				
Female vs. male	0.500	0.855	0.543	1.347				
Smoking history 2/3/4/5 vs. 1	0.463	0.827	0.498	1.373				
Alcohol history Yes vs. no	0.493	0.835	0.498	1.398				
Lymph node Yes vs. no	0.004	0.411	0.226	0.750	0.01	0.438	0.234	0.82
Clinical stage III/IV vs. II/I	0.561	1.311	0.526	3.270				
TXLNA expression High vs. low	0.050	1.802	1.000	3.247	0.103	1.635	0.906	2.951

## Data Availability

The data used to support the findings of this study are available from the corresponding author upon request.

## Supplementary Materials

Supplementary Materials Supplementary Figure 1: the TXLNA gene expression in PAAD patients with different grades. The histogram indicates the expression level of the TXLNA gene at different neoplasm histologic grades (G1, G2, G3, G4, and GX) in patients with PAAD in the TCGA. The values were expressed as the mean ± S.E.M. A one-way analysis of variance (ANOVA) followed by the Tukey's test for post hoc comparisons was used for the statistical evaluation.
