# Supplementary material for: High TXLNA Expression Predicts Favourable Outcome for Pancreatic Adenocarcinoma Patients
Source: Biomed Res Int. 2020 Feb 25;2020:2585862. doi: 10.1155/2020/2585862 (PMC7060861; doi:10.1155/2020/2585862)
Supplement: Supplementary Materials — Supplementary Figure 1: the TXLNA gene expression in PAAD patients with different grades. The histogram indicates the expression level of the TXLNA gene at different neoplasm histologic grades (G1, G2, G3, G4, and GX) in patients with PAAD in the TCGA. The values were expressed as the mean ± S.E.M. A one-way analysis of variance (ANOVA) followed by the Tukey's test for post hoc comparisons was used for the statistical evaluation. [file 2585862.f1.pdf]

Supplementary Figure 1

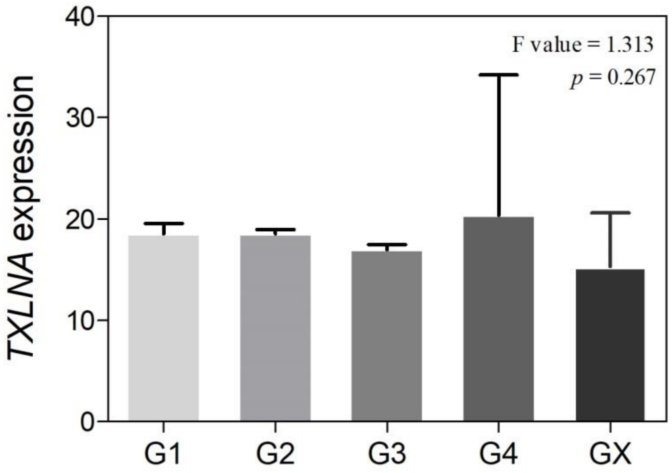

Supplementary Figure 1. The *TXLNA* gene expression in PAAD patients with different grades. The histogram indicates the expression level of *TXLNA* gene at different neoplasm histologic grades (G1, G2, G3, G4 and GX) in patients with PAAD in the TCGA. The values were expressed as the mean ± S.E.M. A one-way analysis of variance (ANOVA) followed by the Tukey's test for post hoc comparisons was used for the statistical evaluation.
